# A case report of diet elimination guided by food specific IgG4 achieved clinical cure for eczematous external otitis with finger paronychia

**DOI:** 10.1097/MD.0000000000039605

**Published:** 2024-09-06

**Authors:** Yuteng Du, Boyun Yang, Wo Yao, Yongbin Zhu, Xuxia Tang, Huiying Wang

**Affiliations:** a Department of Allergy and Clinical Immunology, The Second Affiliated Hospital Zhejiang University School of Medicine, Hangzhou, China; b Department of Emergency Medicine, Hangzhou Third People’s Hospital, Hangzhou, China; c Department of Otolaryngology, The Second Affiliated Hospital Zhejiang University School of Medicine, Hangzhou, China; d Department of Otolaryngology, Zhejiang Provincial Hospital of Traditional Chinese Medicine, Hangzhou, China.

**Keywords:** allergy, diet elimination, eczematous otitis externa, food specific IgG4, treatment

## Abstract

**Rationale:**

Eczematous external otitis (EEO) is the most difficult-to-treat otitis externa, and characterized by the symptoms of inflammation with hypersensitivity of the external ear canal skin. It is acknowledged as a chronic skin inflammation primarily caused by dermatological and allergic reactions. Food allergens are also considered a cause to induce the inflammation. However, the role of food specific IgG4 in this disease is unclear yet.

**Patient concerns:**

A 54-year-old woman complained of recurrent itching of the external auditory meatus for 3 years and nails chapping of hands for 2 years.

**Diagnoses and Interventions:**

She was diagnosed with EEO and underwent the therapeutic strategy as food elimination of egg, milk and wheat, guided by the result of food specific IgG4 together with probiotics on the basis of previous symptom controlling therapy.

**Outcomes:**

After 17 months’ treatment, she was finally free of all the symptoms and the serum IgG4 specific to all foods are under normal limit.

**Lessons:**

To the best of our knowledge, it is the first report revealing the clinical significance of food specific IgG4 in EEO, and the successful treatment with diet elimination guided by food specific IgG4 threw a new light on the clinical management of refractory EEO.

## 1. Introduction

Eczematous external otitis (EEO) is the most difficult-to-treat otitis externa, which is a common disease with a lifetime prevalence estimated at 10%.^[1]^ It is characterized by the symptoms of inflammation such as itching, redness, discharge, desquamation, and so on, with all forms of hypersensitivity of the external ear canal skin.^[1]^ The recurrent attack of acute inflammation could lead to the chronic changes of ear canal skin thickening.^[2]^ The itching and scaling often causes the discomfort and impacts the life quality severely.

Treatment of EEO usually focuses on suppressing the acute flare-up of inflammation.^[3]^ Topic antibiotics and ear-drop of steroids are the first-line treatment, since a secondary infection often occurs and promotes the deterioration.^[4]^ Administration of steroid ear drops is the conventional strategy, and most of them are effective for short term usage. A number of clinical investigations provide the clinical evidence of the efficacy of topic reagents of hydrocortisone,^[5]^ budesonide,^[6]^ fluocinolone acetonide,^[7]^ and pimecrolimus.^[8]^ However, the critical problem lying under the treatment of EEO is the frequent relapsing of the inflammation. Whatever antibiotics or steroids only mitigate the acute inflammation, but cannot prevent the future exacerbation. And long term administration of antibiotics and steroids also brings new problems such as drug resistance or telangiectasia, cutaneous thinning, and atrophy, and other steroid complications.^[9]^ New developed reagents such as honey ear-drop seem helpful to reduce these adverse reactions,^[10]^ whereas, it did not resolve the foresaid fundamental problem. So it is pivotal to find the underlying factors that induce the inflammation, and block the correlative pathway.

The pathogenesis of EEO is not clear yet. However, it is acknowledged as a chronic skin inflammation primarily caused by dermatological and allergic reaction, or the symptoms of atopic dermatitis presented on the ears.^[11]^ Thus, it usually accompanied with skin eczematous inflammation in other place. Allergic reaction to contact allergens such as different metal accessories containing nickle, shampoos, hair dyes, etc, is considered to play important roles in provoking the symptoms. However, some studies indicated that food allergens are also a pathogenic factor.^[12]^ So the detect and avoidance of the culprit foods might be helpful to prevent the inflammation from the initial stage.

Hereby we present an interesting case of EEO with finger paronychia correlated to food specific IgG4, who achieved the clinical cure after diet elimination of the pathogenic foods. From this case, we extend our acknowledgments of the etiology of EEO from a systemic view including the influence of life habits and food intakes, as well as the comprehensive management with add-on food elimination on the basis of conventional medication.

## 2. Case report

A 54-year-old woman complained of recurrent itching of the external auditory meatus for 3 years and nails chapping of hands for 2 years. The itching with skin desquamation occurred on bilateral external auditory meatus with sustaineous white exudates. She had to undertake ear canal lavage every month with 5% polyvinyl pyrrolidone solution. Two years ago, her fingers became red and swollen with nails chapping and solidity. She used topic therapy of vitamin E and urea cream without significant improvement. She had the medical history of 10 years’ allergic rhinitis with nasal spray of steroids as needed, 8 years’ chronic urticaria but under complete remission. Her mother had drug allergy and seasonal dermatitis.

**Figure 1.**
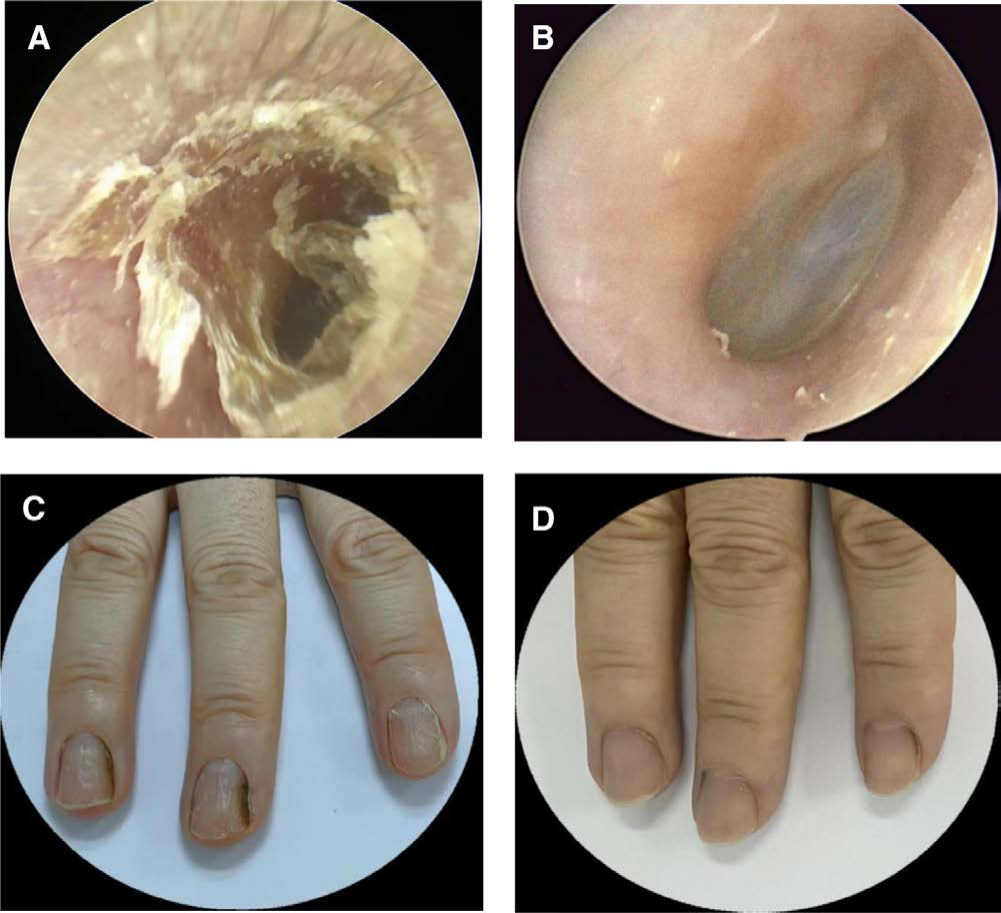
The image of external auditory meatus and fingers.

Physical examination on visit revealed obvious exudates and skin desquamation of her ear canals with redness of finger and chapped nails (Fig. 1). No rash could be seen on skin. Lab examination showed that the results of blood test including white cell differential counts, liver and renal function, immunoglobulins and complements, antinuclear antibody, and cytokines (interleukin-4,5,6,13,10) are within normal ranges. However, erythrocyte sedimentation rate (ESR) is elevated at 30 mm/h. Serum total IgE was at 286 IU/mL (the normal range is < 100 IU/mL) with IgE specific to mixed grass pollens at 0.58 IU/mL and to ovalbumin (OVA) at 0.36 IU/mL. Serum level of milk specific IgG4 (milk-IgG4) was 547.0 U/mL together with egg-IgG4 at 392.9 U/mL, and wheat-IgG4 at 308.5 U/mL, the normal range of these specific IgG4s is < 250 U/mL.

**Figure 2.**
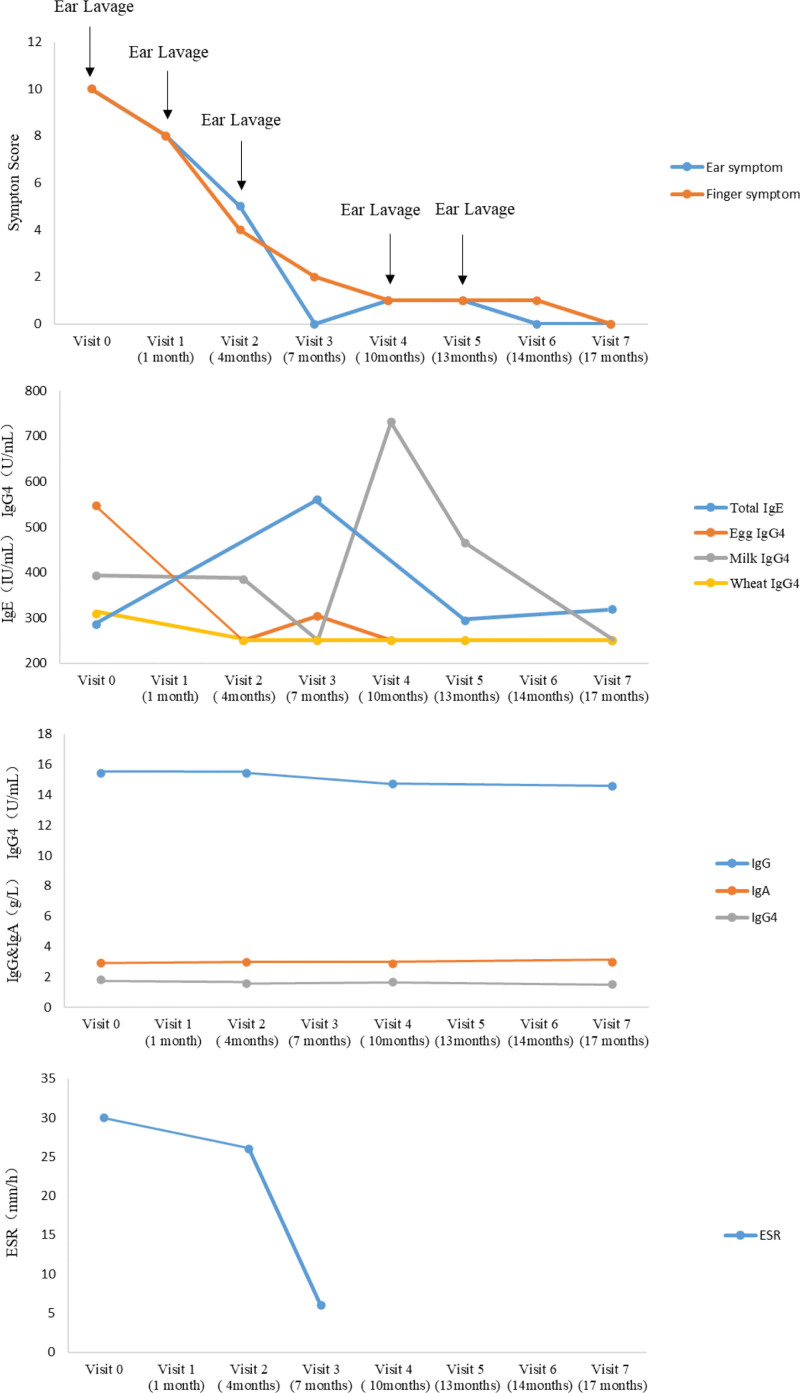
Treatment course and the changes of clinical symptoms and serum index.

She was diagnosed as EEO and suggested to avoid pathogenic foods that contained egg, milk, and wheat, on the basis of the previous symptom-controlling therapy. She also accepted an add-on treatment with probiotics (eN-Lac Plus capsule®) that contain *Lactobacillus paracasei* LP33, *Lactobacillus fermentum* GM090, and *Lactobacillus acidophilus* GMNL-185 (GenMont Biotec Inc.) at the dosage of 1 pill every night. One month later, the symptoms of itching and skin desquamation and nails chap improved obviously. Then she kept follow-up regularly every 3 months under the combined treatment of the allergists and ENT doctors simultaneously with the monitor of her serum index. We used the visual analogy scoring to assess the symptoms at each visit to illustrate the improvement of her clinical manifestation (Fig. 2). The exudation of ear canal on the first visit was set up at 10 score, and the score of each follow-up visit was set according the improvement degree of the exudation and itching. The finger swollen was also scored similarly. On visit 3 (4 months), the exudates decreased dramatically with the obvious reduction of the finger swollen, and ESR dropped to 6 mm/h within the normal range. She stopped the ear canal lavage on visit 5. The patient completed the 7 visits with the duration of 17 months and finally was free of all of the symptoms without topic medications. During the treatment, her serum total IgE increased first and then dropped down to 319 IU/mL finally. The serum IgG4 specific to all foods were under normal limit <250 U/mL. The milk-IgG4 fluctuated during the treatment for the seldom intakes of milk. Serum IgG, IgA, total IgG kept at a stable level within normal range, and was 14.55g/L, 2.98g/L, 1.55 g/L, respectively, at last visit. She was considered as the clinical cure for the disappearance of symptoms without medication.

## 3. Discussion

In present clinical investigation, we reported a case of refractory EEO with successful treatment of diet elimination guided by food specific IgG4 for the first time, to the best of our knowledge. We observed the clinical progress of the patient with comprehensive assessment of clinical symptoms and serum immunologic index, and revealed the clinical significance of food specific IgG4 in EEO.

EEO as a topic dermatitis arising on ear skin, has a complex pathogenesis including genetic factors, environmental factors, and surely microbiotic factors. The skin barrier impairment and allergen contacts cause the allergic reaction commonly, with the hyper-response to different allergens and often present as allergic contact dermatitis.^[13]^ Skin prick test or total IgE level both confirm its atopic characteristics. Based on the chronic inflammatory condition, bacterial infections often provoke the acute exacerbation.^[14]^ And fungi infection otherwise is common for the chronic persistent inflammation, with typical pathogens as *Aspergillus* and *Candida*.^[2]^ In this patient, the diagnosis of chronic EEO is proved, with the developed finger paronychia, part of skin chronic inflammation. Her past medical history and family history reveals an atopy background, which further confirms this diagnosis. On the initial visit, the patient demonstrated an obvious systemic inflammation status with the ESR at 30 mm/h. The increased total IgE level also illustrated the allergic status. Previous treatment of this patients is the regular larvage of the external tunnel of ears and topic medications of steroids and antibiotics, which relieved the symptoms but could not suppress the inflammation. Finding the underlying factors that cause the persistent inflammation is important for the patient.

The serologic test gave us some clues. The serum food specific IgEs were almost negative excepted OVA-IgE, which was a little bit higher than the normal range at 0.36 IU/mL. The clinical manifestation as no immediate reaction to eggs excluded the OVA-IgE mediated food allergy. In the contrast, egg, milk, and wheat might be the culprits according to the result of food specific IgG4 test. And the improvement of clinical symptoms accompanied by the drop to normal range of food specific IgG4s confirmed our suspicion.

Food specific IgG4 is conventionally considered as a product of natural exposure without conclusive role in allergic diseases or other diseases.^[15]^ And it was not recommended as the diagnostic tool for food allergy.^[16]^ However, more emerging evidence demonstrates the pathogenic role of food specific IgG4 in allergy and chronic inflammation.^[17]^ Those clinical investigations reveal that the increased food specific IgG4 were associated to multiple allergic diseases such as atopic dermatitis, asthma, eosinophilic esophagitis, food allergy, and so on.^[18–22]^ Our previous study shows that food specific IgG4 is the only predict factor for the prognosis of allergic diseases in children.^[23]^ Po-Jen Liu et al, also reports that 8 week food elimination guided by food specific IgG4 improved the hearing threshold in children with otitis media.^[24]^

The patient had 3 positive specific IgG4 to milk, egg, and wheat at the first visit. So we tried the therapeutic strategy of the diet elimination of milk, egg, and wheat, combined with previous symptom-controlling treatment. The add-on of probiotics is aimed to modulate the immune system via the gut microbiota. And this patient exhibited a dramatically improvement, with the gradually alleviation of the exudation of the external ear canals and mitigation of finger chap till completely cure after 17 months’ treatment. During the treatment with the avoidance of the related foods, egg- and wheat-IgG4 dropped gradually. However, the milk-IgG4 fluctuated significantly. The possible reason is associated with the production of specific IgG4, which is correlated to the intake of foods. Avoidance of the foods will reduce the production and naturally degradation gradually leads the drop of the serum level to the normal range. So the level of those food specific IgG4s usually fluctuate if the diet elimination is not strictly. In our patient, milk-IgG4 fluctuated dramatically for the occasionally intake of milk.

In our investigation, the patient is considered clinical cure for free of symptoms and medications. The diet elimination of milk, egg, and wheat shows a significant role in the progress of the disease with the gradually evanish of the inflammation. That is never achieved by the previous symptom-controlling treatment for several years. Usually, the patient will be allowed to take those foods gradually after 3 months observation without reoccurrence.

In a summary, our study reveals a clinical correlation of the food specific IgG4 to EEO and demonstrates a novel treatment strategy of diet elimination, which throws a new light on the management of refractory EEO. Further studies that exploring the underlying mechanisms and clinical efficacy are needed.

## Acknowledgments

We appreciate the patient and the doctors for their collaboration in this study.

## Author contributions

**Data curation:** Huiying Wang.

**Investigation:** Boyun Yang, Wo Yao.

**Visualization:** Yongbin Zhu.

**Writing – original draft:** Yuteng Du.

**Writing – review & editing:** Xuxia Tang, Huiying Wang.
